# Aluminum

**Published:** 1889-01

**Authors:** 


### Aluminum.

The following article published in the *Lockport American*, is from the pen of Prof. Leonard Waldo, General Manager of the Aluminum Bronze Works in that city.

As aluminum and its alloys are rapidly coming into prominence in many directions in the arts; and also as a material for dental purposes as well, it is given a place here.

This metal has been introduced and is being used to some extent for plates for artificial dentures, and it is desirable that its qualities and properties be fully understood.

Dr. Waldo's experience and scientific knowledge are such that his utterances should receive special attention. And this we ask of the readers of the REGISTER. ED. REG.

ALUMINUM—ITS USES AND COST DISCUSSED AT LENGTH BY PROF. WALDO.

An article on "Costly Aluminum" by "C. M." which originally appeared in the Springfield *Union*, has been reprinted by you and my attention has been called to it. There is so much interest in aluminum and its alloys, especially in Waterbury circles, that I think somebody ought to point out, that the facts in regard to aluminum are more honored in their breach than in their observance in this particular article. To begin with, there is the best authority for spelling and pronouncing the word aluminum [as the *American* said editorially.] In the next place it does not oxidize in the air nor tarnish even at a strong red heat. It is not even attacked by sulphuretted hydrogen or sulphide of ammonium, and in cities where the ordinary gases would soon blacken silver table ware, aluminum would remain bright and untarnished. There is no reason for thinking that common salt, at least table salt, would have the slightest effect upon pure aluminum. It is not attacked by nitric acid nor by sulphuric acid unless it is very stroug. Hydrochloric acid will attack it, but the vegetable acids which we get in ordinary cooking have no effect upon it with the exception that a mixture of acetic acid and common salt will effect it, but to a much less extent than it would effect tin under the same circumstances. There is, however, this difference, that in the case of the aluminum the action of the acid mixtures would be to form a compound of alumina which would not be poisonous, whereas, in the case of tin or copper, the resulting compound would be directly dangerous to human life. The commonest example of the free use and exposure to air of aluminum is found in the beautiful opera and field glasses, always purchased when they can be afforded. The aluminum cap to the Washington Monument could hardly have less exposure to atmospheric influences, and it has no evidence of deterioration.

As for strength, while it does not belong to a group of metals whose prominent quality when chemically pure, is strength, yet it has a tensile strength of 26,800 pounds to the square inch as compared with 16,000 pounds for cast iron and 50,000 pounds for wrought iron. This is comparing it by a sectional area; but if we compare it by taking the greatest length of a bar in feet able to support its own weight we have for the cast iron 5,351 feet, for steel and aluminum each 23,040 feet. So that taking the strength of aluminum in relation to its weight it has about the mechanical value of a steel having 35 tons tensile strength per square inch.

In working aluminum the metal has to be treated by shop methods which are peculiar to itself, but which differ no more than do the methods of working steel and brass.

Its melting point is 100° centigrade higher than was mentioned, and it can be soldered readily if the shop methods are understood by the workmen.

But I think where the article in question is most misleading, is in its want of consideration of the fact that the chief use of aluminum in the arts to-day is not in its pure state, but in its alloys with copper and iron. There are certain definite compounds which aluminum makes with copper and iron which give to these metals properties of the utmost value in the arts, and furnish raw material of a quality and price not reached by any other methods. It is not necessary to more than mention the aluminum bronzes. The best of these bronzes have a beautiful gold color, take a high polish, are very malleable, extremely hard, and have a tenacity of the best steel. The aluminum brasses are easily made to double the mechanical qualities of the ordinary brasses. For instance, the companies furnishing aluminum brass sell it in ingots with a tensile strength up to and exceeding 60,000 pounds per square inch, and furnish the aluminum bronzes up to and exceeding 90,000 pounds per square inch. If we take cast brass at 23,000 pounds and gun bronze at 39,000 pounds (Trantwine's "Engineer's Pocketbook" 1885) we get a clear indication of the effect of aluminum on brasses and bronzes. I think this question is one of marked importance to the Naugatuck valley brass and copper mills. Those interested should consult in proof of this the official letter of William H. Harris, the Chief Engineer of the United States navy, giving the impartial comparisons of the ordinary bronzes with some aluminum bronzes and brasses regularly made and sold by the Cowles Company in the United States.

The percentages of aluminum added to copper have ranged from $1\frac{1}{4}$ per cent. to 10 per cent. and if we take the market price even as high as $3 per pound for the contained aluminum, and this is higher than large consumers are now obliged to pay, it will be seen that the additional cost ranges between $4\frac{1}{2}$ and 28 cents per pound above the price of copper. This latter alloy is a highly ornamental one and the difference in price can often be well afforded. When we come to the action of aluminum on iron, results are even more remarkable. A recent number of the *Iron Age* contains an exhaustive paper on this subject, read at the last meeting of the American Association for the Advancement of Science, held at Cleveland. This paper, which has been widely copied both here and in Europe, shows that, as a result ot an exhaustive series of foundry experiments conducted at the Michigan stove works, Detroit, even percentages of aluminum as small as one-twentieth of 1 per cent. have a marked effect upon iron castings for the better. The castings are practically free from blow holes, have a closer grain, a greater strength, and in general the additional cost of say one-sixth of 1 cent per pound is a most profitable one for the iron founder to incur. I doubt whether there is a single large iron or steel casting establishment in the United States which is not now considering the use of aluminum in iron or already using it.

For most commercial uses the addition of the small per centage of silicon to the aluminum is a decided advantage and the market for commercial aluminum at $2 a pound could not but be a very large one. The commercial value of aluminum is so great, and the technical difficulties of its production so hard to surmount, that there have been, as the writer of the article referred to says, a number of impostures in the field. But the amount of imposture is no greater than average human nature warrants, or than crops up in technical electricity, or at the beginning of any distinct advance in the industrial arts when accurate knowledge is confined to the few. On the cost of production and selling price the writer of the article referred to is much misinformed. Commercially pure aluminum can be bought in the London market at 20 shillings per pound, and for use in copper, brass, or iron it can be bought in the United States in its appropriate alloy at considerably less than that sum.

The largest aluminum works in the world are now being erected in England, and as to the cost of production I can only say that it is already less than the $2 a pound referred to as the lowest price at which it can be produced.

